# Self-administered Fecal Microbial Transplants—What Could Possibly Go Wrong?

**DOI:** 10.1093/crocol/otaa025

**Published:** 2020-04-17

**Authors:** Laura E Raffals

**Affiliations:** Division of Gastroenterology and Hepatology, Mayo Clinic, Rochester, Minnesota, USA

Fecal microbial transplant (FMT) has proven to be an effective therapy for *Clostridioides difficile* infections with efficacy rates greater than 90%.^1^ It is believed that the success of this treatment is due to the ability of FMT to restore the colonic microbial community to a healthy state. Although the etiology of inflammatory bowel disease (IBD) is not entirely understood, it is proposed that in genetically susceptible individuals, environmental triggers alter the immune response which is driven in part by the intestinal microbiota. It seems logical that resetting the intestinal microbiota would in turn reset the immune response, serving as an effective treatment for IBD. Despite the theoretical rationale behind the use of FMT for the treatment of IBD, clinical trials have been disappointing.^2–7^

In this issue, Sandler and colleagues describe the use of FMT and patient-reported outcomes associated with FMT in the Crohn’s Colitis Foundation IBD Partners cohort. This cohort includes over 15,000 individuals who have identified as having a diagnosis of IBD. A 13-question survey inquiring about the use of FMT was administered to 5430 IBD Partners participants and 67.2% completed the survey. History of FMT was low in this group, with only 51 individuals reporting prior FMT. FMT was more common in the ulcerative colitis patients compared to Crohn’s disease. The use of 5-ASA, immunomodulators, and biologics was not significantly different in those patients who had prior FMT compared to those who did not undergo FMT, although the FMT recipients were more likely to have used probiotics, rectal steroids, budesonide, and systemic steroids. Despite this, patient-reported disease activity was similar between those individuals who received FMT and those who did not.

FMT was used to treat concomitant *C. difficile* in 22 patients and was delivered by colonoscopy or nasogastric tube in 19/22 of these patients. In contrast, in those patients who did not have *C. difficile* as an indication for FMT, self-administration of FMT and the use of FMT without physician direction were common (72.4% and 79.4%, respectively). For those patients who received FMT for *C. difficile*, 14/22 (63.6%) reported complete relief of symptoms compared to only 3/29 (10.3%) patients who underwent FMT for other reasons. Although there are limitations to this study given the self-reported nature of this IBD cohort, it highlights the willingness of some patients to turn to nonmainstream treatments for their IBD without the guidance of a physician and without clear evidence of efficacy.

More than ever, patients are empowered consumers of their health care experience and are taking a more active rather than passive role in their disease management. It is rare to meet an IBD patient who has not researched treatment strategies, both traditional and nontraditional, before an office visit. IBD patients are frequently turning to nonconventional treatment approaches with reports of 30%–50% of IBD patients using complementary and alternative medicines; yet less than 50% of patients using alternative therapies share this information with their treating physician.^8–10^ Many “natural” or nonmainstream therapies have not undergone rigorous testing to ensure the benefit of treatment outweighs potential risks. Unfortunately, risks associated with FMT have recently been realized. In June 2019, the United States Food and Drug Administration issued a safety communication regarding FMT and potential risk of transmission of multidrug-resistant organisms following reports of 2 immunocompromised patients who developed infections with extended-spectrum beta-lactamase-producing *Escherichia coli* leading to the death of one of the patients. The FMT capsules were derived from the same donor and subsequent analysis revealed that more than 20 additional recipients of FMT capsules derived from that donor were also found to have the same strain of *E. coli* in their post-FMT stool specimens.^11^

There are many risks our IBD patients face including IBD-related complications and adverse effects from medications used to treat their disease. As gastroenterologists, we have learned to guide our patients to understand the absolute risks associated with the treatments we recommend with the intent to maximize their benefit and improve the quality of our patients’ lives. This study highlights the practice of some patients, even if a minority, to seek treatments outside of the mainstream that are associated with risks that may or may not be fully recognized. In the setting of self-administered FMT, our patients, many of whom are immunocompromised, are exposing themselves to a stool that could contain a number of pathogens.

We must be willing to take the time to ask our patients what approaches they take to treat their disease which are not prescribed by us so that we can ensure our patients are informed. Our patients’ faith in natural remedies does not need to be at odds with our recommendations. It is our responsibility to express our shared mission to improve the health of our patients and educate them with the data we have at hand and help them choose safe and effective treatment strategies.

## Notes

Sources of support: None.

Funding disclosures: None.
